# Self-assessed visual function outcome in cataract surgery: minimum important difference of the Catquest-9SF questionnaire

**DOI:** 10.1186/s40662-022-00318-x

**Published:** 2022-12-06

**Authors:** Magnus Grimfors, Mats Lundström, Maria Kugelberg

**Affiliations:** 1 Department of Clinical Neuroscience, St. Erik Eye Hospital, Karolinska Institutet, 171 64 Stockholm, Sweden; 2 Department of Clinical Sciences, Ophthalmology, Faculty of Medicine, Lund University, Lund, Sweden

**Keywords:** Cataract surgery, Minimum important difference, MID, Patient reported outcome measures, PROM, Visual function, Catquest-9SF, Questionnaire, Cataract

## Abstract

**Background:**

The purpose of this study was to study the minimum important difference (MID) of the Catquest-9SF questionnaire in cataract surgery.

**Methods:**

A nationwide multi-center prospective randomized study was conducted using the Swedish National Cataract Register and the Catquest-9SF questionnaire. Randomized patients (n = 400) who had completed the Catquest-9SF before surgery and three months after surgery were sent an anchor question on self-assessed change in visual function after cataract surgery 14 days after the postoperative Catquest-9SF. Rasch analysis was performed on the preoperative and postoperative Catquest-9SF questionnaires, and the patients were dichotomized with regard to their preoperative Rasch score. The MID range of the two groups was calculated based on the anchor question, and the anchor question based MID was then estimated in a scatter plot. The MID was also estimated based on distribution by calculating Cohen’s effect size.

**Results:**

The analyses included 231 patients who had completed the Catquest-9SF on both occasions as well as the questionnaire with the anchor question. The group with better preoperative visual function had an anchor question based MID of − 0.5 and a Cohen’s effect size based MID of − 1.07. The group with worse preoperative visual function had an anchor question based MID of − 1.80 and a Cohen’s effect size based MID of − 1.46.

**Conclusion:**

This article contributes detailed knowledge of the MID of Catquest-9SF, enabling even more accurate high-quality evaluation of the outcome and benefit of cataract surgery worldwide.

## Background

Cataract is not only a common disease, but also remains the leading cause of blindness in the world [1]. This is also true in high-income countries. Worldwide, significant resources are spent treating the large and steadily increasing number of patients with cataract [2]. The situation calls for continued accurate high-quality evaluation of the outcome and benefit of cataract surgery.

**Fig. 1:**
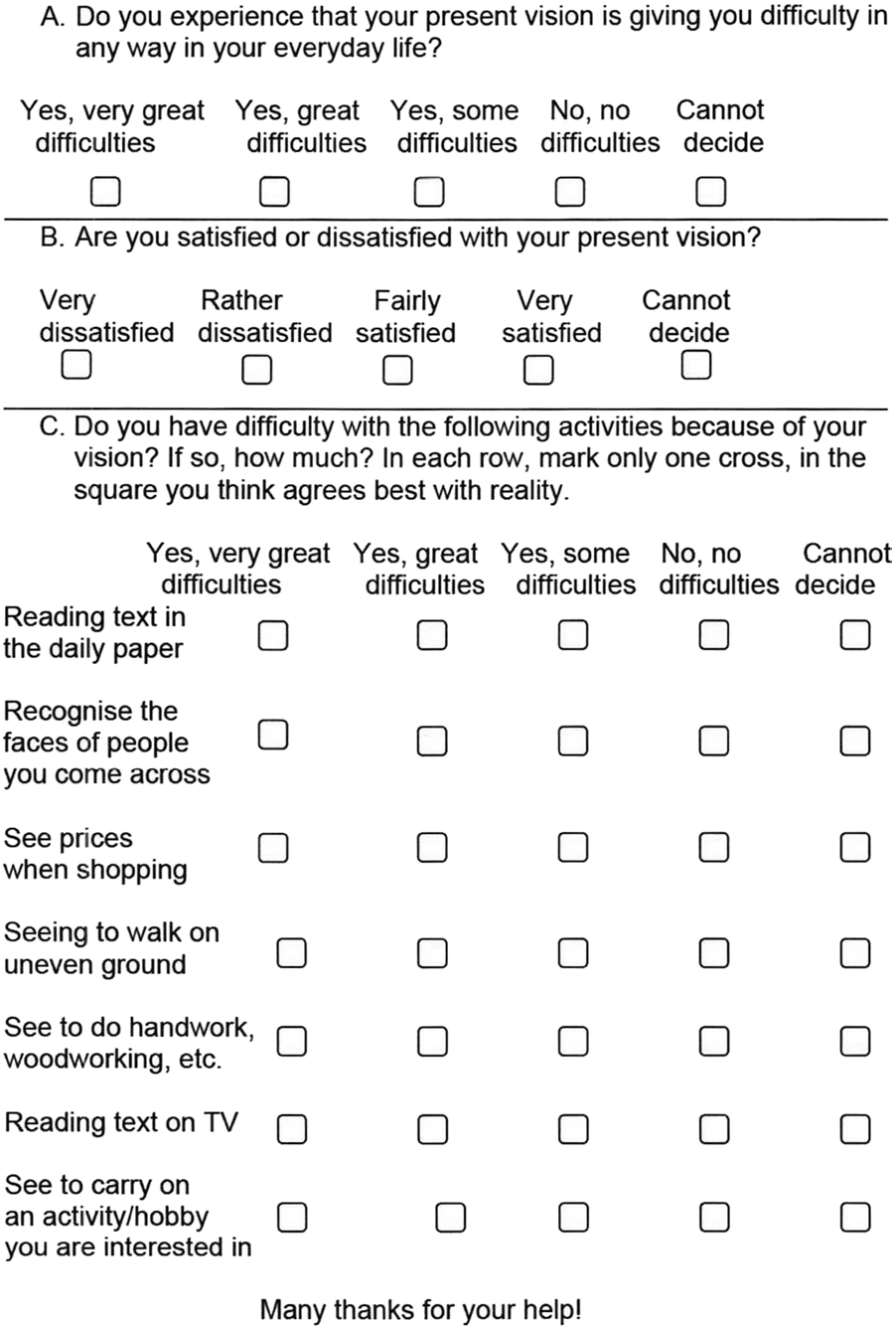
The English version of the Catquest-9SF

Visual acuity is perhaps the most common and accessible measure of vision, but it does not sufficiently reflect the entirety of the complex human visual function. Complete evaluation of the patient’s visual function and the impact of cataract surgery demands a more advanced measure. The Swedish National Cataract Register (NCR) was introduced in 1992 [3], and from 1995 began to use the Catquest questionnaire to collect data on patient-reported visual function [4]. Since then, the questionnaire has been revised using Rasch analysis, resulting in the current version: Catquest-9SF (Fig. 1) [5]. The Rasch model, which is often used to assess psychometric data such as individual abilities, attitudes, and personality traits, is based on probabilistic relationships between persons (e.g., patients) and items (e.g., questions). The ordinal (raw) data are converted to an interval level measurement (a logit unit), which allows assessment of the psychometric properties of the measurement using the Rasch model.

To evaluate a patient’s visual function and the impact of cataract surgery, the Catquest-9SF is completed by the patient before cataract surgery and then again three months postoperatively. The Rasch-revised version has been shown to be highly valid in measuring visual disability outcomes of cataract surgery in several different populations: Swedish [5], Australian [6], German and Austrian [7], Malay and Chinese [8, 9], Italian [10], Spanish [11], Dutch [12], English [13], Danish [14], New Zealander [15], and Vietnamese [16]. A recent systematic review of these validation reports concluded that the Catquest-9SF is a valid and reliable tool to measure visual function in patients with cataract in various populations [17]. In a comparison with other tools, the Catquest-9SF was found to be short, highly responsive to cataract surgery, and a good measure of visual function outcomes [18]. In 2017, it was chosen as the recommended tool for version 2.0.1 of the ICHOM Cataracts Data Collection Guide [19]. Moreover, a review of the quality of 17 patient-reported cataract outcome instruments concluded that the Catquest-9SF demonstrated superior psychometric properties as well as high responsiveness, and should be considered the recommended instrument for cataract surgery [20]. A recent test-retest study also showed excellent reliability [21].

Earlier studies using the Catquest-9SF to assess the association between ocular comorbidity and self-assessed visual function after cataract surgery [22] and the association between intraoperative difficulties and self-assessed visual function after cataract surgery [23] showed several significant associations. Using extensive data from the NCR, often with more than 10,000 patients in each study, makes even rather small associations statistically significant. However, it remains unclear if these associations are of clinical importance to the patient. Therefore, the aim of the current study was to investigate the minimum important difference (MID) of the Catquest-9SF.

## Methods

### Study design

The study was conducted as a prospective randomized multi-center study using the NCR, one questionnaire with a single anchor question, and the Catquest-9SF questionnaire. For the sample size calculation, we used 100,000 Monte-Carlo simulations. It has already been shown that the difference in preoperative and postoperative Rasch score is normally distributed, and so the simulations were based on a multivariate normal distribution with a correlation of 0.7. A regression analysis was performed for each simulation, with the anchor question as explanatory category variable and the difference in Rasch score as the outcome. With 240 patients, a significant difference in Rasch score between the response options “somewhat better” and “much better” was seen in 100% of the simulations and between “somewhat better” and “no change” in 72% of the simulations. We considered this to give enough precision to associate a positive change in Rasch score with a positive change according to the anchor question.

### Intervention

Randomly selected patients (n = 400) from the nationwide NCR scheduled for cataract surgery in the first or second eye completed the Catquest-9SF before surgery and three months postoperatively. The Catquest-9SF questionnaire includes nine questions: two on global vision-related difficulties and satisfaction with vision, and the remaining seven on difficulties performing specific activities (Fig. 1). Each question has five response options, including “cannot decide”. Fourteen days after completing the postoperative Catquest-9SF questionnaire, the anchor question on self-assessed change in visual function after cataract surgery was distributed to the patients via a questionnaire containing a single question: “How do you experience your present vision compared to your vision before the cataract surgery?” This had five response options: “much better”, “somewhat better”, “no difference”, “somewhat worse”, and “much worse”. The study was approved by the Stockholm Regional Ethical Review Board (reference number 2017/130-31/2) and adhered to the tenets of the Declaration of Helsinki.

### Statistics

Rasch analysis was performed using version 4.2.0 of Winsteps (Chicago, IL, USA) on the preoperative and postoperative Catquest-9SF questionnaires (n = 231), and the patients were dichotomized with regards to their preoperative Rasch score. The concept of MID in this study is based on the change in Rasch score from before to after surgery. The room for improvement is smaller for patients with few problems (low Rasch score) before surgery compared with those having more problems before surgery (high Rasch score). In such circumstances it is reasonable to use different MID values depending on the baseline score. Therefore, we chose to dichotomize the preoperative Rasch score, and we used the lower percentile (25%) as the dividing point. Patients with Rasch scores of − 4.91 to − 1.50 were placed in Group 1, and those with scores of − 1.49 to 5.73 in Group 2. The Rasch score change was calculated by subtracting the preoperative Rasch score from the postoperative Rasch score. MID range of the two groups (mean Rasch score change) was calculated on the basis of the anchor question. The anchor question based MID was then estimated in a scatter plot. Version 27 of IBM SPSS (SPSS Inc., Chicago, IL, USA) was used for statistics. The MID was also estimated on the basis of distribution via Cohen’s effect size (d) [24], which is calculated by dividing the difference of the mean postoperative Rasch score and the mean preoperative Rasch score by the standard deviation of the preoperative Rasch score: d = (mean postop Rasch − mean preop Rasch)/SD. The MID is half of Cohen’s effect size.

## Results

Of the 400 originally included patients, 234 responded (59%) and 231 were included in the final analyses. Of the 234 patients who completed both occasions of the Catquest-9SF and the questionnaire with the anchor question, three were excluded due to giving an inconclusive answer to the anchor question. The inconclusive answers did not stick to the given response options but instead text that could not be interpreted. The analyses therefore included 231 patients with a mean age of 74 years, of whom 60% were female. The mean age of the excluded patients (n = 169, of whom n = 166 were non-responders) was also 74 years, and 62% were female.

**Fig. 2:**
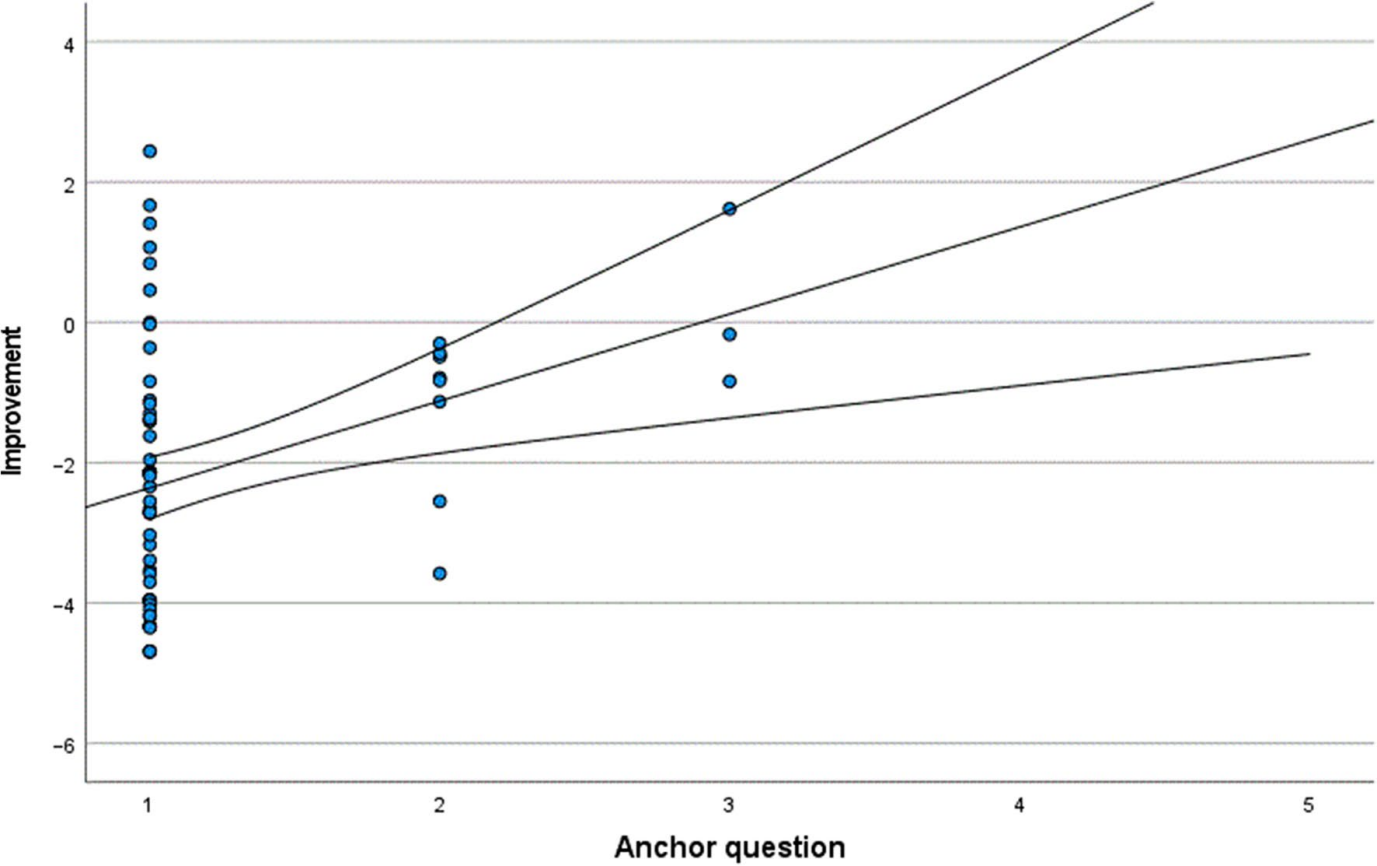
Scatter plot showing improvement in Rasch score and answers to anchor question in Group 1. Group 1 comprises patients with a preoperative Rasch score of − 4.91 to − 1.50. The central line in the scatterplot is a line of best fit using the least squares method and the upper and lower lines represent 95% confidence intervals

**Fig. 3:**
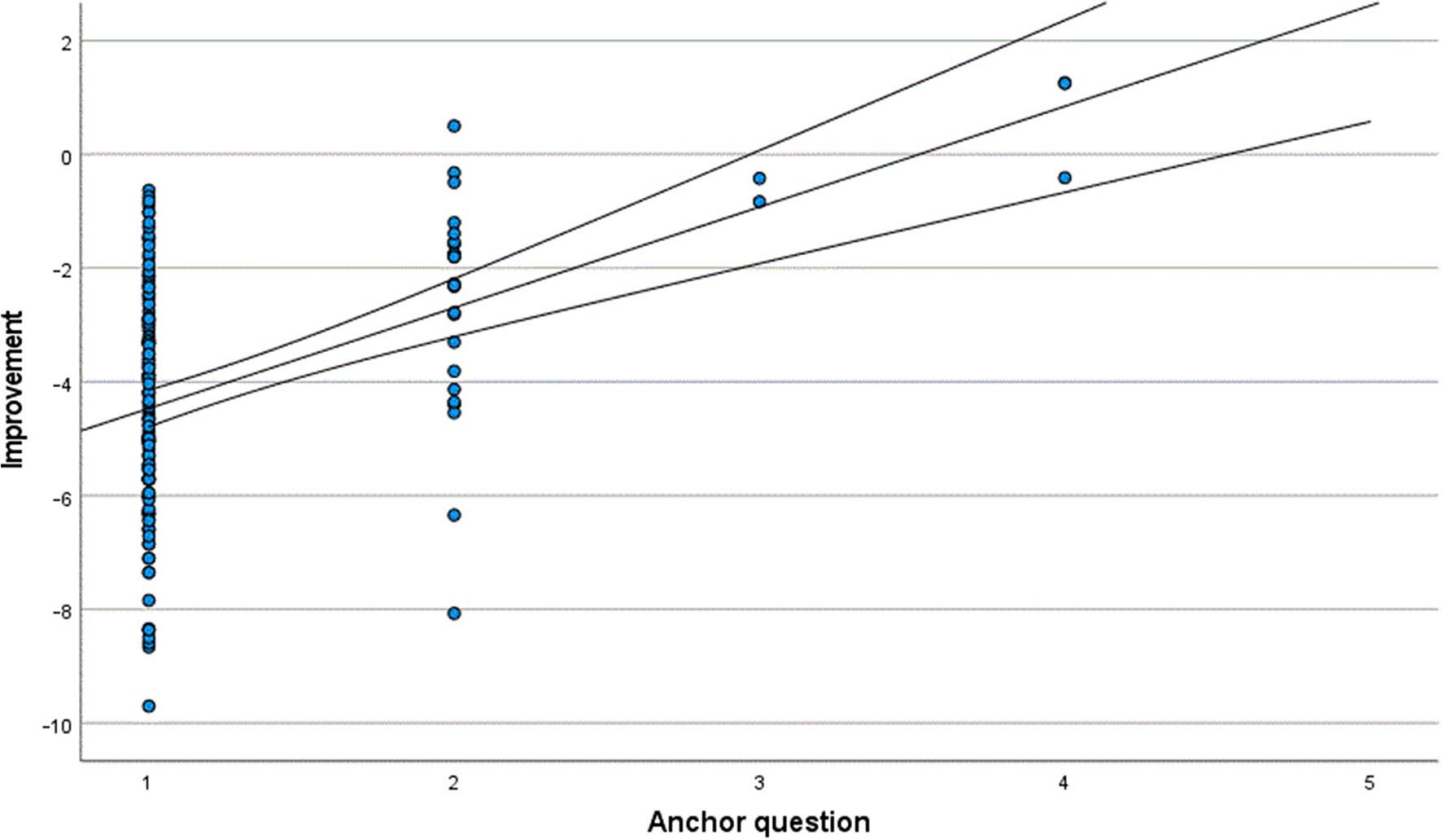
Scatter plot showing improvement in Rasch score and answers to anchor question in Group 2. Group 2 comprises patients with a pre-operative Rasch score of − 1.49 to 5.73. The central line in the scatterplot is a line of best fit using the least squares method and the upper and lower lines represent 95% confidence intervals

The results of the anchor question for Groups 1 and 2 are shown in Tables 1 and 2, respectively. The MID range for Group 1 was estimated to be between 0.2033 and − 1.1756, based on the mean Rasch score change in patients who felt there was “no difference” in their vision and patients who felt they had “somewhat better” vision after cataract surgery. The MID range for Group 2 was estimated to be between − 0.6250 and − 2.6892. A more precise MID of − 0.5 in Group 1 and MID of − 1.80 in Group 2 was then calculated from the scatter plot where the trend line met the x value of 2.5, corresponding to the midpoint between answer 2 (“somewhat better”) and answer 3 (“no difference”) (Figs. 2, 3). The MID estimated on the basis of distribution by calculating Cohen’s effect size was − 1.07 for Group 1 and − 1.46 for Group 2. The different estimations of MID are shown in Table 3.

**Table 1:** Mean Rasch score change in Group 1

Answer anchor question	Mean Rasch score change	Number of patients (n)	Percentage of patients (%)
1 (Much better)	− 2.3551	57	83
2 (Somewhat better)	− 1.1756	9	13
3 (No difference)	0.2033	3	4
4 (Somewhat worse)	–	0	0
5 (Much worse)	–	0	0
All	− 2.09	69	100

Group 1 comprises patients with a preoperative Rasch score of − 4.91 to − 1.50; that is, the group with better preoperative self-assessed visual function

**Table 2:** Mean Rasch score change in Group 2

Answer anchor question	Mean Rasch score change	Number of patients (n)	Percentage of patients (%)
1 (Much better)	− 4.4669	133	82
2 (Somewhat better)	− 2.6892	24	15
3 (No difference)	− 0.625	2	1
4 (Somewhat worse)	0.7	3	2
5 (Much worse)	–	0	0
All	− 4.0604	162	100

Group 2 comprises patients with a preoperative Rasch score of − 1.49 to 5.73; that is, the group with worse preoperative self-assessed visual function

**Table 3:** Estimations of minimum important difference (MID) in each group

Means of MID estimation	Group 1	Group 2
Anchor question	− 0.5	− 1.80
Cohen’s effect size	− 1.07	− 1.46
Approximation of true MID (mean of the above)	− 0.79	− 1.63

Group 1 comprises patients with a preoperative Rasch score of − 4.91 to − 1.50; that is, the group with better preoperative self-assessed visual function. Group 2 comprises patients with a preoperative Rasch score of − 1.49 to 5.73; that is, the group with worse preoperative self-assessed visual function

## Discussion

The aim of the current study was to investigate the MID of the Catquest-9SF. One previous study using the Catquest-9SF to assess the association between ocular comorbidity and self-assessed visual function after cataract surgery [22] showed several significant associations. Using extensive data from the NCR, often with more than 10,000 patients makes even rather small associations statistically significant. However, it remains unclear if these associations are of clinical importance to the patient when MID is not known. For example, in the previous study [22], assessing the effect of glaucoma on changes in subjective visual function after cataract surgery, glaucoma turned out to be significantly associated with changes in patient-reported visual function with a logit change of − 0.355. The MID estimations from the current study (e.g., − 0.79 as in Group 1) can be used to determine if it is likely that the presence of glaucoma is of clinical importance to the patient. In this case, the change of − 0.355 being less than the MID of − 0.79, indicates that the presence of glaucoma is probably of minor clinical importance.

The MIDs estimated by the two methods in the current study were similar. Using two separate methods to estimate the MID of Catquest-9SF could be argued to add to the accuracy of the estimation. A previous study including 846 patients who completed the Catquest-9SF before and after cataract surgery showed an effect size of − 1.87, which corresponds to a MID of − 0.935 [25]. This value is similar to the results of the current study.

A strength of this study is the expected finding that Rasch score change after cataract surgery differed depending on the baseline Rasch score and the subsequent dichotomization into separate groups. The room for improvement is smaller for patients with few problems (low Rasch score) before surgery compared with those having more problems before surgery (high Rasch score). In such circumstances, it is reasonable to use different MID values depending on the baseline score. Without the dichotomization into two separate groups, the estimated MID would be imprecise. The rather large difference in the estimated MID of the two groups indicates the importance of taking the baseline Rasch score into account when evaluating the Rasch score change after cataract surgery. As in the example above, when assessing the effect of glaucoma on changes in subjective visual function after cataract surgery, it is of great importance to adjust for the baseline Rasch score in the study population when assessing if it is likely that the presence of glaucoma is of clinical importance to the patient.

As mentioned, one reason for dichotomizing is that patients with better preoperative visual function already have a low Rasch score, with limited scope for improvement. The analysis of the two groups showed that Group 1 (with a better preoperative visual function) had a significantly lower MID than Group 2 (with a worse preoperative visual function) even though the change in Rasch score was almost twice as large in Group 2. This is an interesting finding. One can argue that patients with a rather good visual function before surgery are just as likely as their worse-functioning counterparts to self-assess their visual function as having improved after surgery, even though the expected improvement measured in logits is lower compared to patients with worse preoperative visual function. Regardless, this new information of MID variation being dependent on the Rasch baseline allows a more precise evaluation of self-assessed visual function after cataract surgery.

Of the 400 originally included patients, 234 responded (59%) and 231 were included in the final analyses. The excluded patients were of similar age and gender as the included patients.

It is worth discussing whether the number of included patients is a weakness. A limitation of the study’s statistical power is the proportionally few patients experiencing only a mild improvement or no change of the visual function, as the vast majority considered their postoperative visual function to be “much better” than before the surgery. This is consistent with previous knowledge that a majority of cataract patients experience a marked improvement of visual function after surgery. Under these circumstances, it is difficult to construct an anchor question with many patients experiencing only a mild improvement or no change of the visual function, which could give this study better statistical power. However, our finding that two separate methods produced similar estimations for the MID of Catquest-9SF could be argued to add to the accuracy of the estimation.

## Conclusion

This article contributes detailed knowledge of the MID of Catquest-9SF, enabling even more accurate high-quality evaluation of the outcome and benefit of cataract surgery worldwide.

## Data Availability

The datasets used and analyzed during the current study are available from the corresponding author on reasonable request.
